# Generalized pustular psoriasis (von Zumbusch) flares successfully treated with Spesolimab. Report of two cases and review of the literature^☆^

**DOI:** 10.1016/j.abd.2024.10.010

**Published:** 2025-05-06

**Authors:** Raquel Steglich, Felipe Saboia, Lincoln Helder Zambaldi Fabricio, Eoda Steglich, Anber Tanaka

**Affiliations:** a Department of Dermatology, Universidade Regional de Joinville, Joinville, SC, Brazil; b Department of Dermatology, Hospital Universitário Evangélico Mackenzie, Curitiba, PR, Brazil

Dear Editor,

Generalized pustular psoriasis (GPP) is a severe, rare autoinflammatory disease characterized by recurrent widespread erythema and sterile pustules that can coalesce to form lakes of pus.^1^ Its prevalence in Brazil is estimated to be between 0.7 and 0.9 cases per 100,000 inhabitants.^2^ The treatment of GPP is challenging, as traditional antipsoriatic medications, including biologics, have limited evidence of efficacy in GPP. Recently, the anti-IL36R biologic, spesolimab, has shown promise.^1^ This study reports the first two Brazilian cases of GPP treated with spesolimab.

## Case 1

**Fig. 1:**
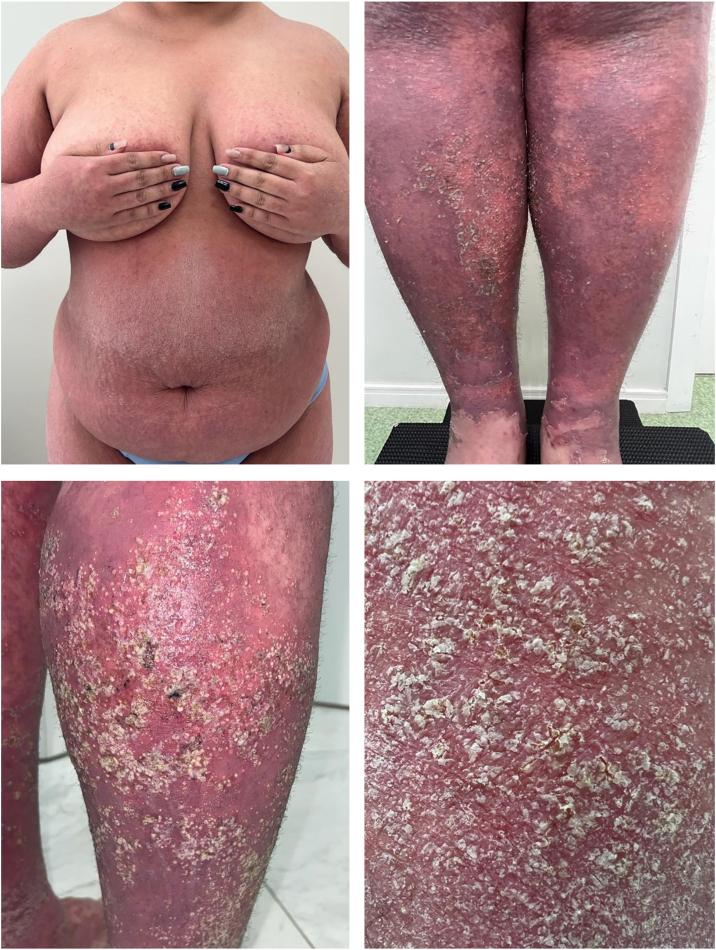
Patient in flare before the infusion ‒ GPPASI = 54.2 and GPPGA = 4.

**Fig. 2:**
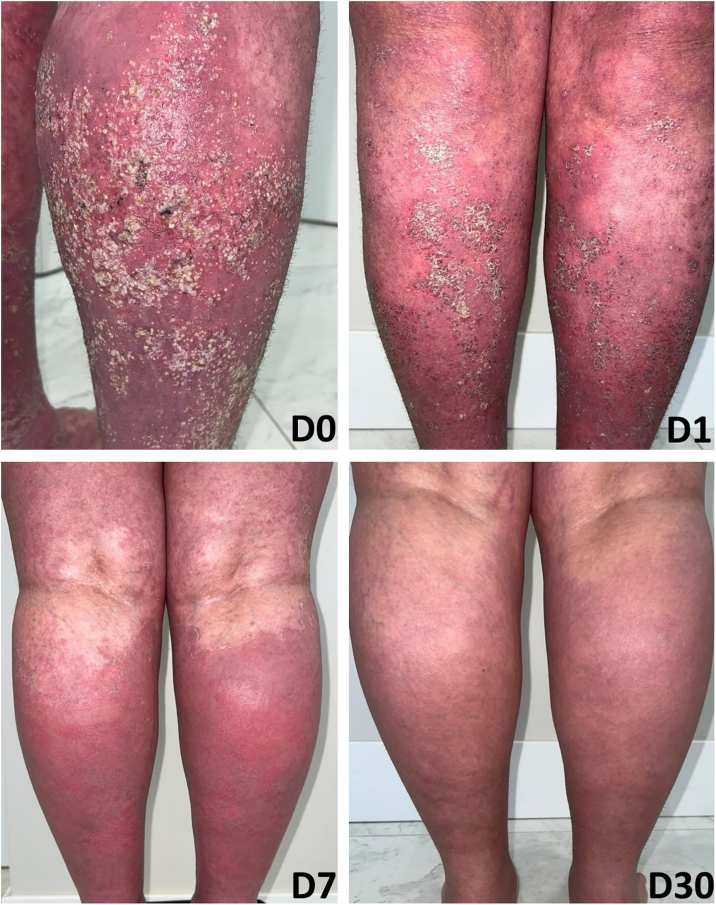
Sequential images before the infusion; one day; one week; and one month after the infusion.

A 27-year-old woman reported a history of recurrent flares of erythematous lesions associated with pustules since the age of 11. She was diagnosed with GPP at the age of 21, during the second trimester of pregnancy, when she developed a generalized condition with systemic symptoms requiring hospitalization, and GPP was confirmed by skin biopsy. At that time, cyclosporine was initiated, resulting in complete control of the lesions. During outpatient follow-up, cyclosporine was discontinued in search of alternatives for maintenance treatment. The use of methotrexate showed improvement in the condition; however, localized pustular lesions persisted. The induction of the medication risankizumab resulted in a new flare, which was contained by the use of cyclosporine. At 27-years-old, the patient experienced a new flare, with diffuse erythema, desquamation, numerous pustules, pain, edema, malaise, and significant impairment of quality of life. On this occasion, an infusion of 900 mg of spesolimab was administered, resulting in an extremely rapid response in both cutaneous and systemic symptoms. Within 24 hours, all pustules had dried up, and there was a sensation of reduced edema, and remission of pain, with only the crusts of the pustules and milder erythema compared to the previous day persisting. At the follow-up visit one week later, there were no pustules, only erythema in the areas where the lesions had been most intense (Fig. 1, Fig. 2). The patient maintained complete remission of the lesions, with no lesions observed during 16 weeks of follow-up.

## Case 2

**Fig. 3:**
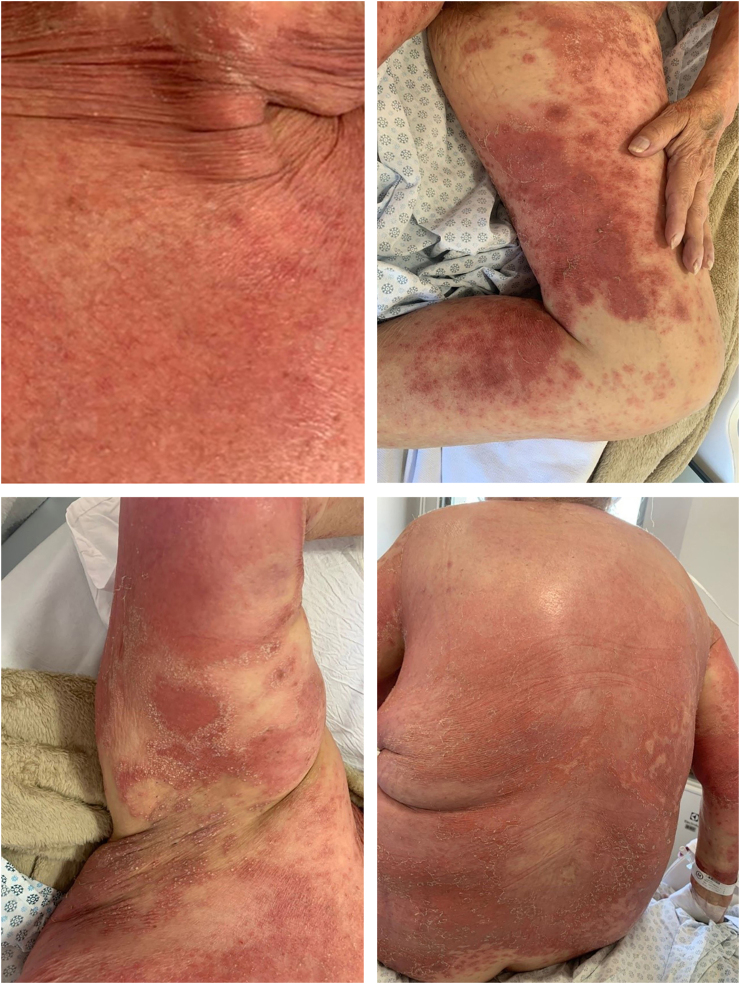
Patient during hospitalization in flare – GPPASI = 23.1 and GPPGA = 3.

**Fig. 4:**
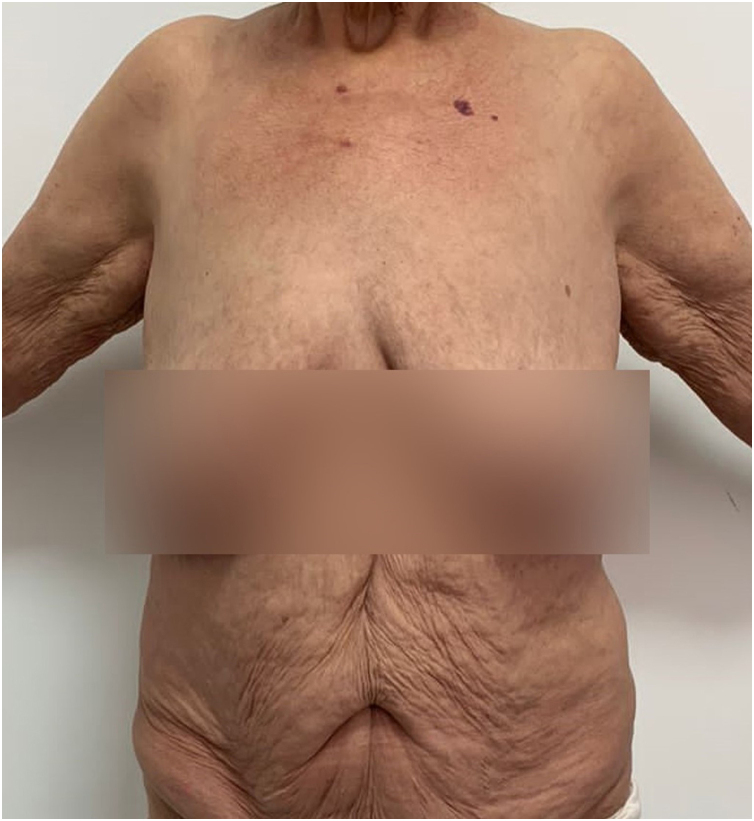
One week after 2 doses of spesolimab administered at weekly intervals.

A 66-year-old woman presented with complaints of erythematous lesions with pustules, progressing to fever and dissemination of the lesions over the past week. She reported previous episodes with similar characteristics, which were treated with systemic corticosteroids and antibiotics. Based on the ERASPEN diagnostic criteria and the skin biopsy, she was diagnosed with GPP.^3^ Acitretin, cyclosporine, and corticosteroids were used in sequence, without achieving clinical control. In an attempt to manage the condition, 900 mg of spesolimab was administered intravenously. One week after the infusion, there was a partial response (GPPASI = 10.2 and GPPGA = 2), and a second dose of 900 mg of spesolimab was administered. The patient showed a rapid response to the medication in the following week (GPPASI = 3 and GPPGA = 1) and maintained complete remission of the lesions, with no flares during 12-weeks of follow-up (Fig. 3, Fig. 4).

## Discussion

GPP is characterized by a sudden eruption of sterile pustules on erythematous base with systemic symptoms.^4^ Disease flares can be idiopathic or triggered by external factors such as infections, discontinuation of systemic corticosteroids, pregnancy, and use of certain immunobiological drugs.^5^, ^6^ The pathogenesis involves the exaggerated activation of the IL-36 Receptor (IL-36R). The cytokines IL-36α, IL-36β, and IL-36γ activate IL-36R, while the IL-36 Receptor antagonist (IL-36Ra) modulates the action of IL-36 cytokines. These cytokines stimulate the inflammatory response, and activation of neutrophils, macrophages, dendritic cells, and T-cells.^4^ The IL-36RN gene encodes the IL-36Ra protein that contributes to suppressing the action of IL-36 cytokines. Mutation of this gene triggers exaggerated signaling of IL-36 cytokines. Mutations in this gene have been identified in both isolated cases^7^ and familial cases^8^ of GPP. Spesolimab, a novel anti-IL36R medication, has shown efficacy in treating GPP flares and was approved by ANVISA in March 2023. The Effisayil-1 trial showed that spesolimab cleared all lesions within a week in 54% of the patients vs. 6% in the placebo group).^1^ A review showed that there is limited evidence regarding the use of other biological drugs to treat GPP compared to spesolimab.^9^ This drug represents a safe and efficient alternative compared to traditional treatments such as immunosuppressants and retinoids.

## Conclusion

GPP is a severe dermatosis that can be confused with other diseases, making diagnosis and treatment challenging. Traditional therapies can cause significant side effects. Spesolimab, an anti-IL36R medication, emerges as a promising and safe alternative in the treatment of GPP, providing rapid improvement of cutaneous and systemic symptoms. It is suggested that further research and the inclusion of spesolimab in established treatment protocols for generalized pustular psoriasis be considered.

## Disclaimer

Boehringer Ingelheim was given the opportunity to review the manuscript for medical and scientific accuracy as it relates to Boehringer Ingelheim substances, as well as intellectual property considerations.

## Financial support

None declared.

## Authors’ contributions

Raquel Steglich: Approval of the final version of the manuscript; intellectual participation in propaedeutic and/or therapeutic management of studied cases; preparation and writing of the manuscript⁠; manuscript critical review.

Felipe Saboia: Critical literature review; data collection, analysis and interpretation; preparation and writing of the manuscript.

Lincoln Helder Zambaldi Fabricio: Approval of the final version of the manuscript; intellectual participation in propaedeutic and/or therapeutic management of studied cases.

Eoda Steglich: approval of the final version of the manuscript; intellectual participation in propaedeutic and/or therapeutic management of studied cases.

Anber Tanaka: Approval of the final version of the manuscript; intellectual participation in propaedeutic and/or therapeutic management of studied cases; manuscript critical review.

## Conflicts of interest

Raquel Steglich: Abbvie – lecturer; Boehringer-Ingelheim – lecturer; Janssen – advisory board, lecturer.

Felipe Saboia: None.

Lincoln Fabricio: Abbvie – lecturer, investigator; Bayer – lecturer; Bioderma – lecturer; Biolab – lecturer; Galderma – lecturer; Hypera Pharma – lecturer; Isdin – lecturer; Janssen – lecturer, investigator; La Roche-Posay – lecturer; LEOPharma – lecturer; Pfizer – lecturer; GSK – lecturer; Novartis – lecturer, investigator; Sanofi – lecturer, investigator; Ache – lecturer.

Eoda Steglich: None.

Anber Tanaka: Abbvie – advisory board, consultant, lecturer, investigator; Boehringer-Ingelheim – advisory board, consultant, lecturer, investigator; Eli-Lilly – advisory board, consultant, lecturer, investigator; Janssen – advisory board, consultant, lecturer; Leo Pharma – advisory board, consultant, lecturer; Novartis – advisory board, consultant, lecturer; investigator; UCB Biopharma – advisory board, consultant, lecturer; Pfizer – lecturer, advisory board; Sanofi – lecturer.
